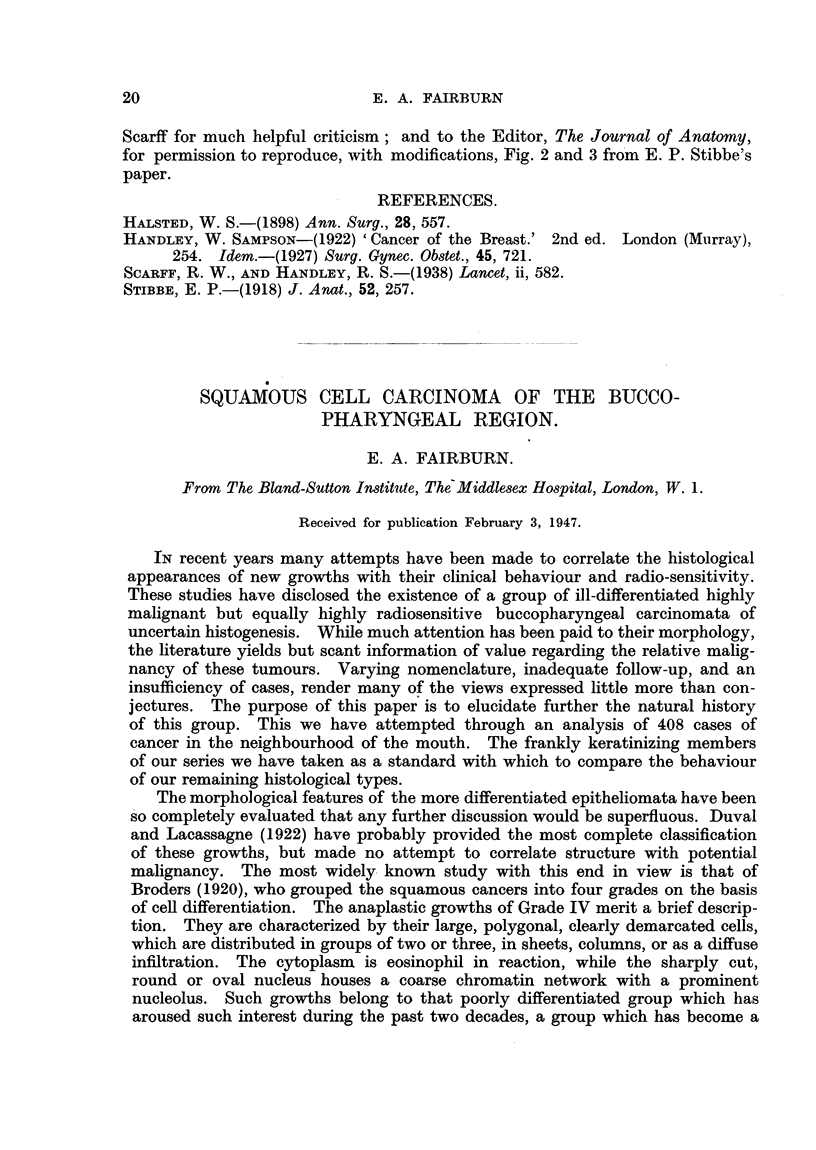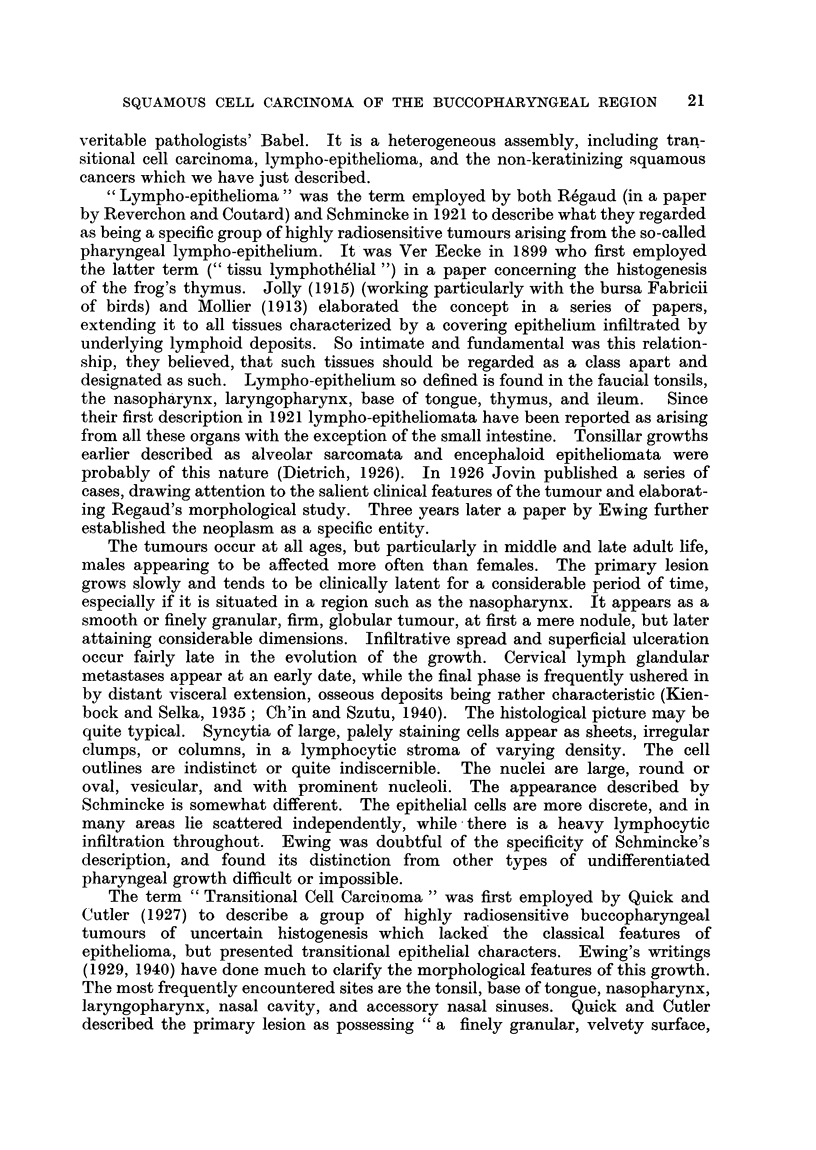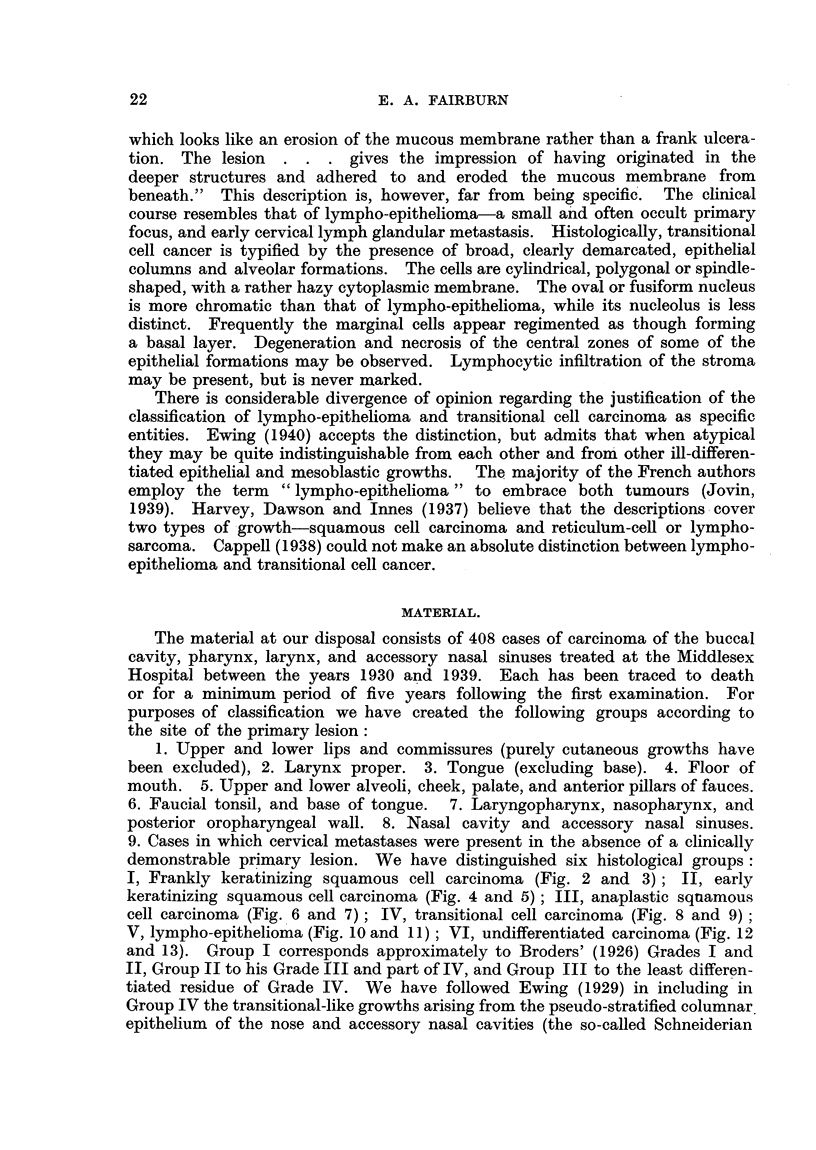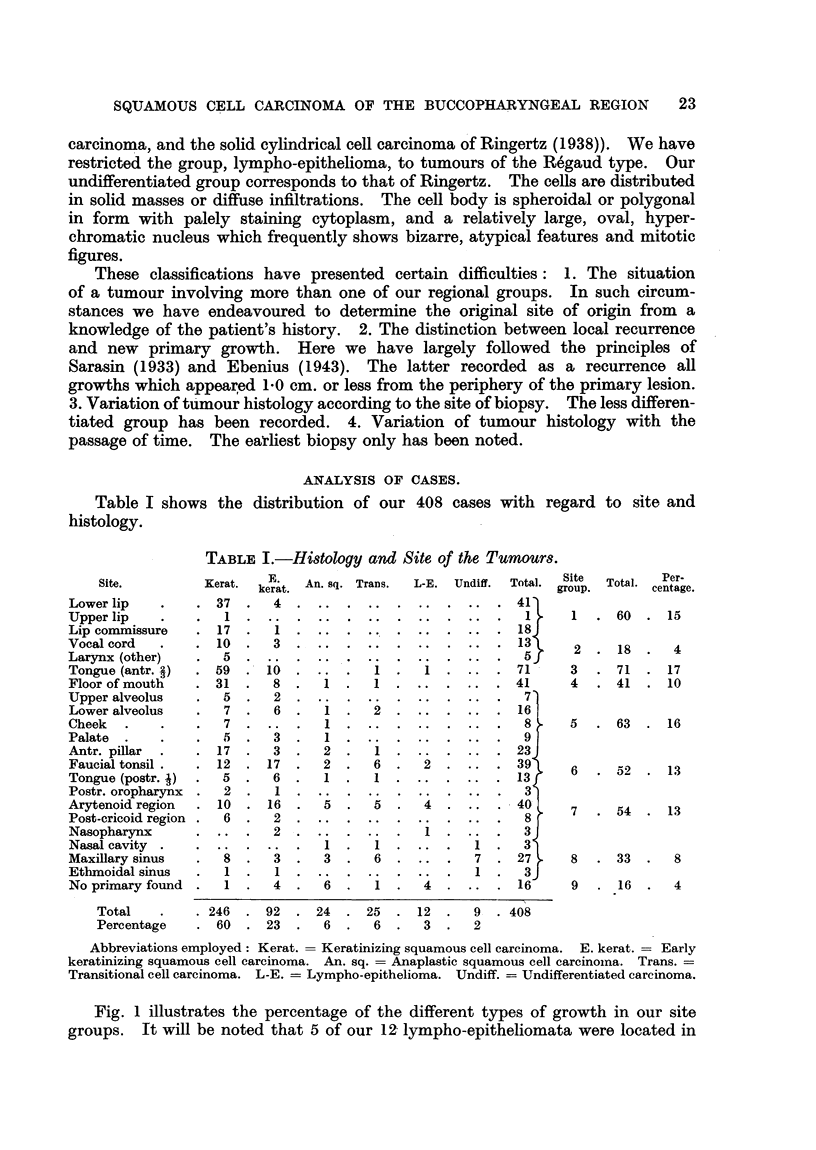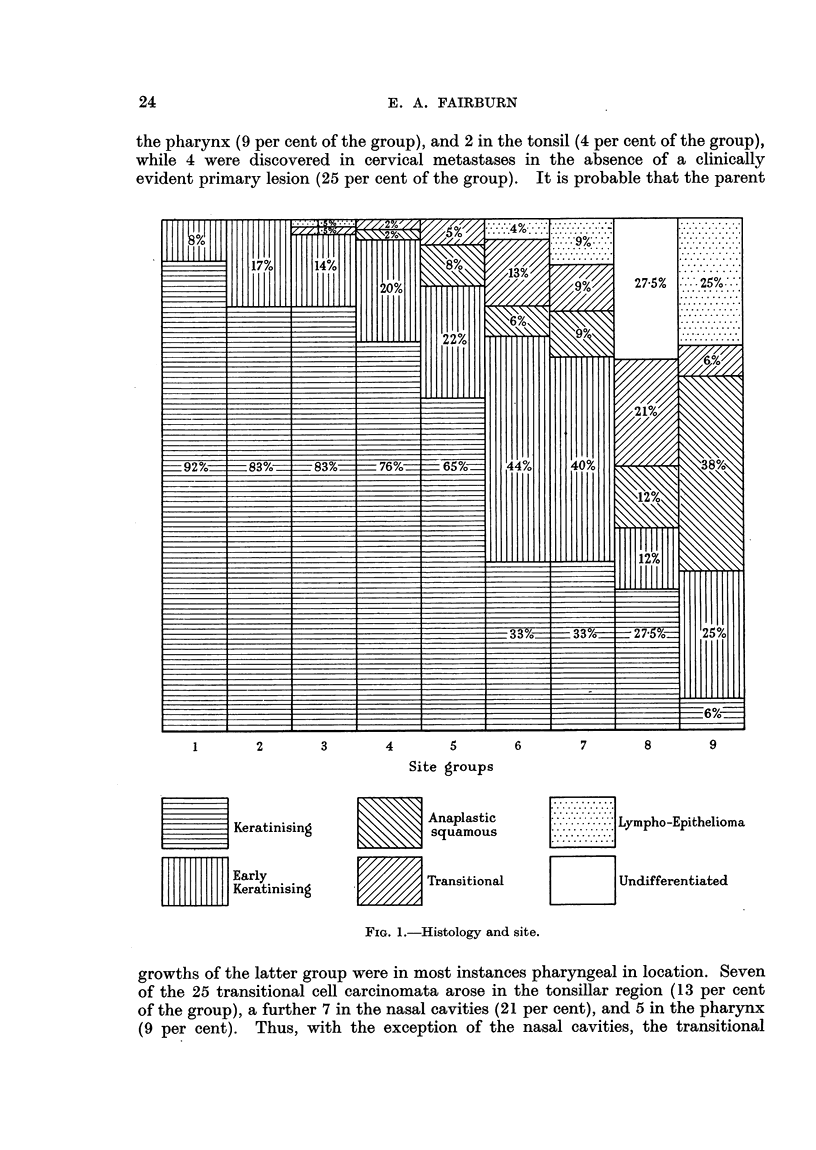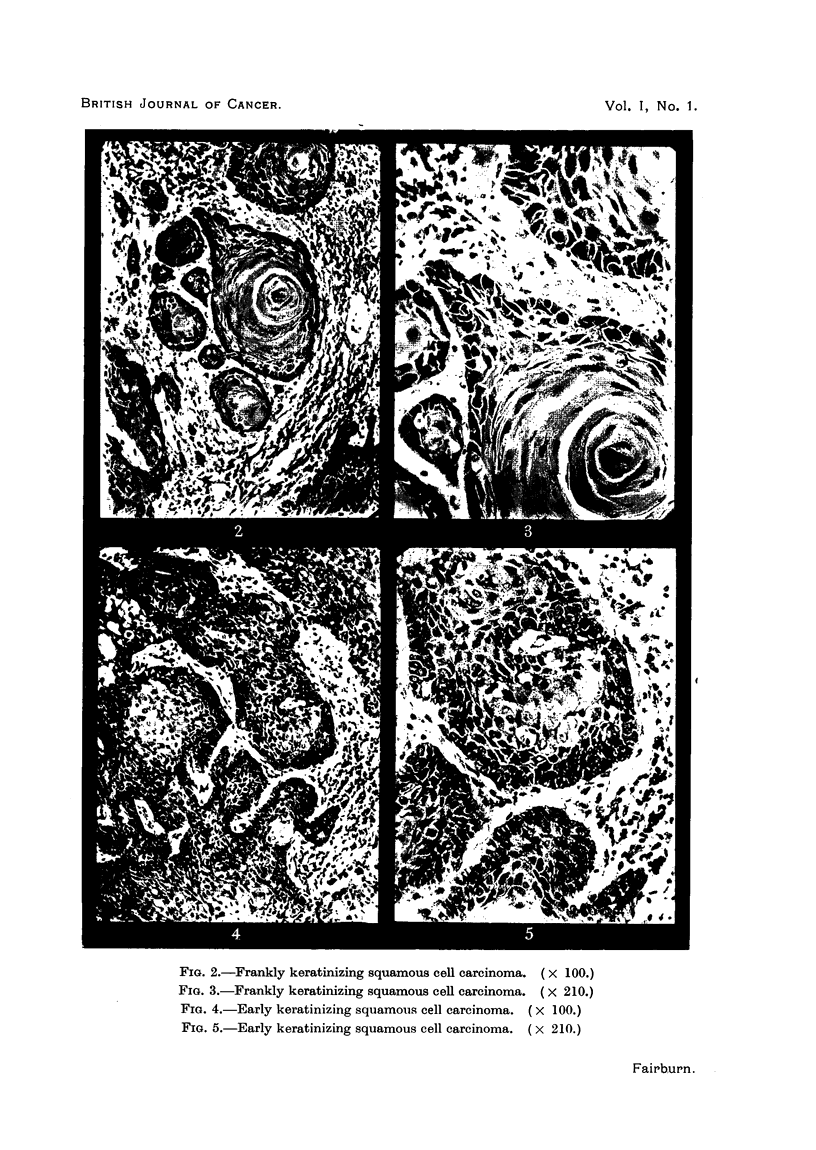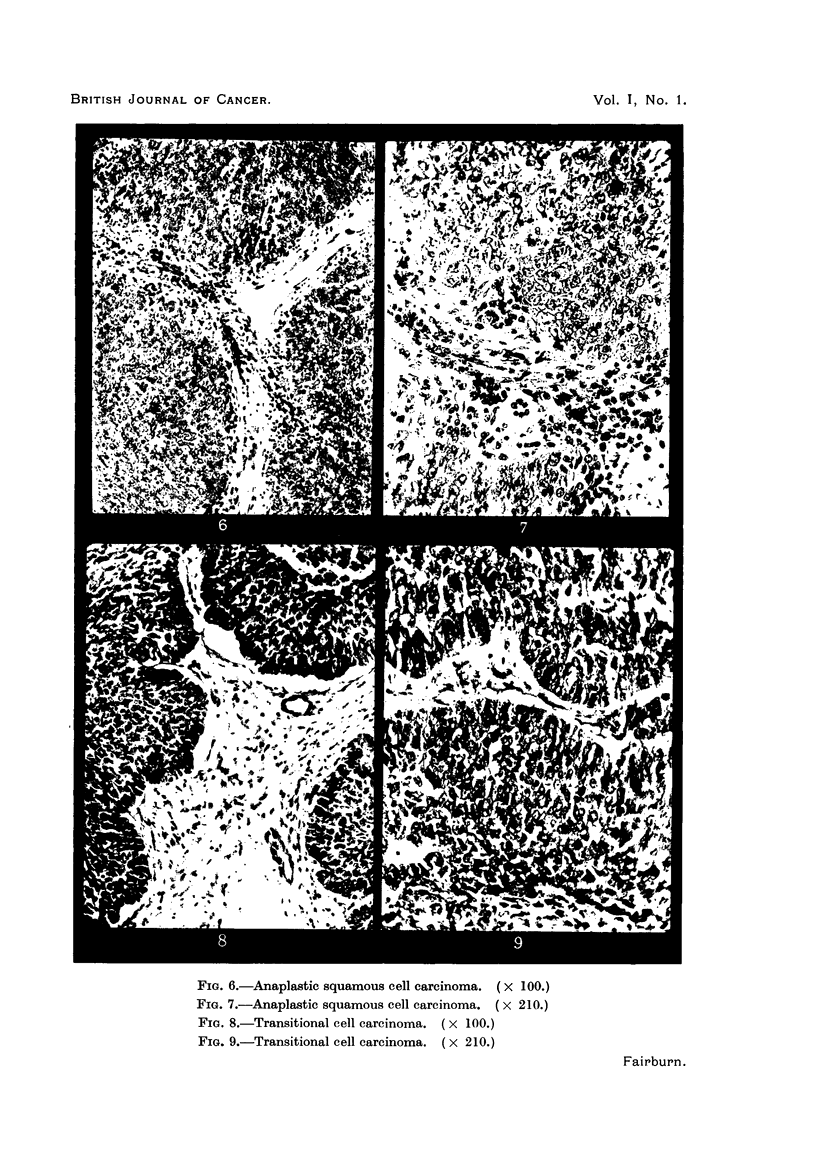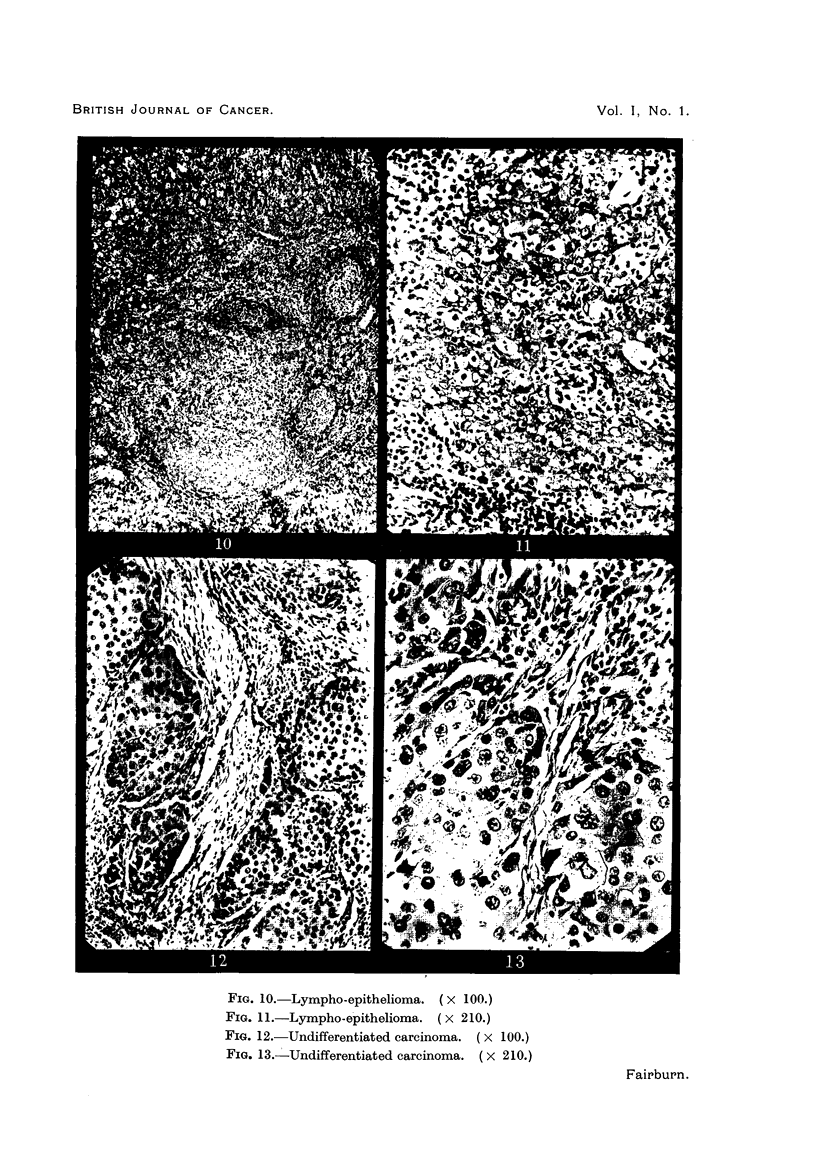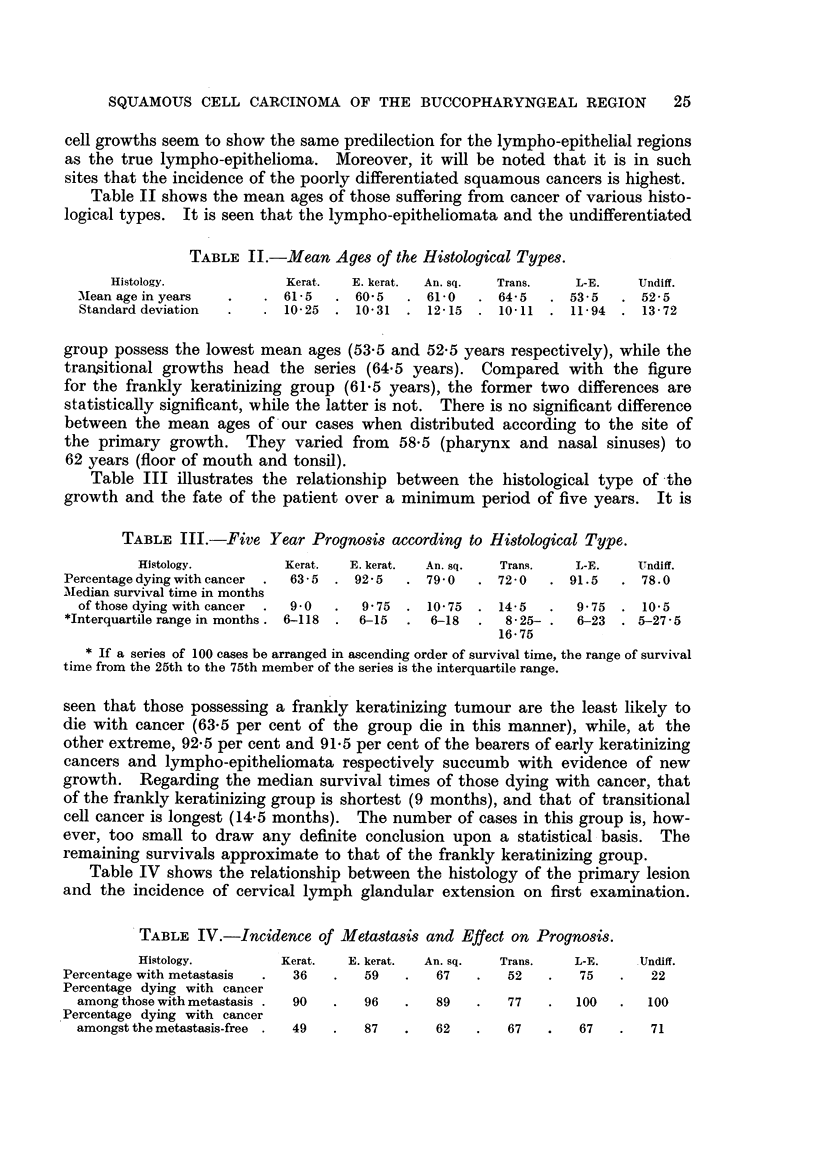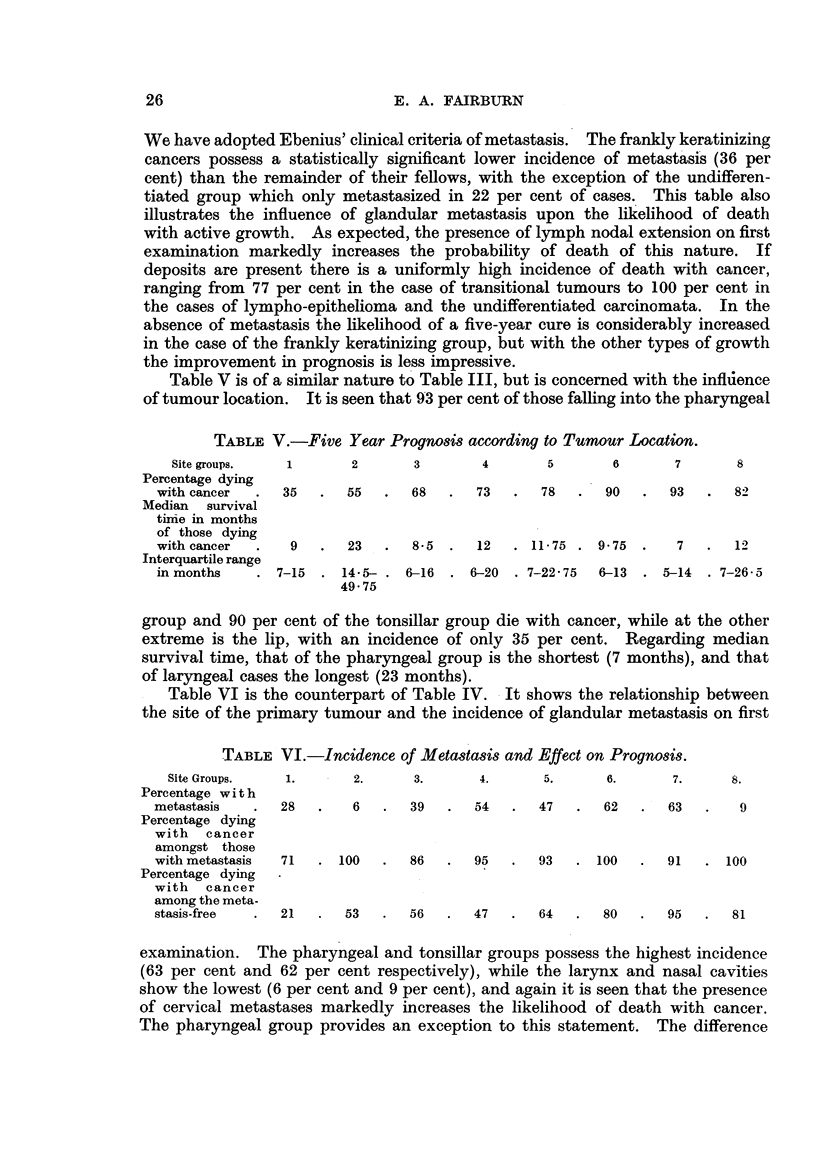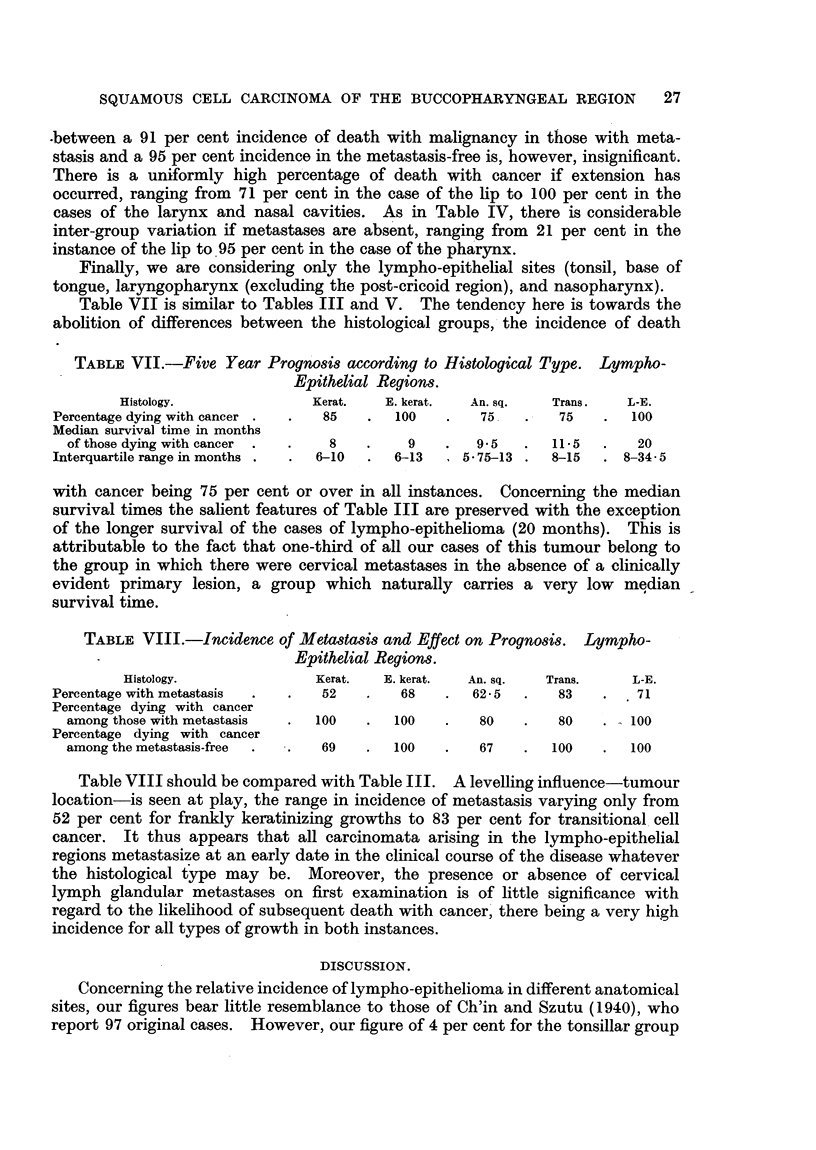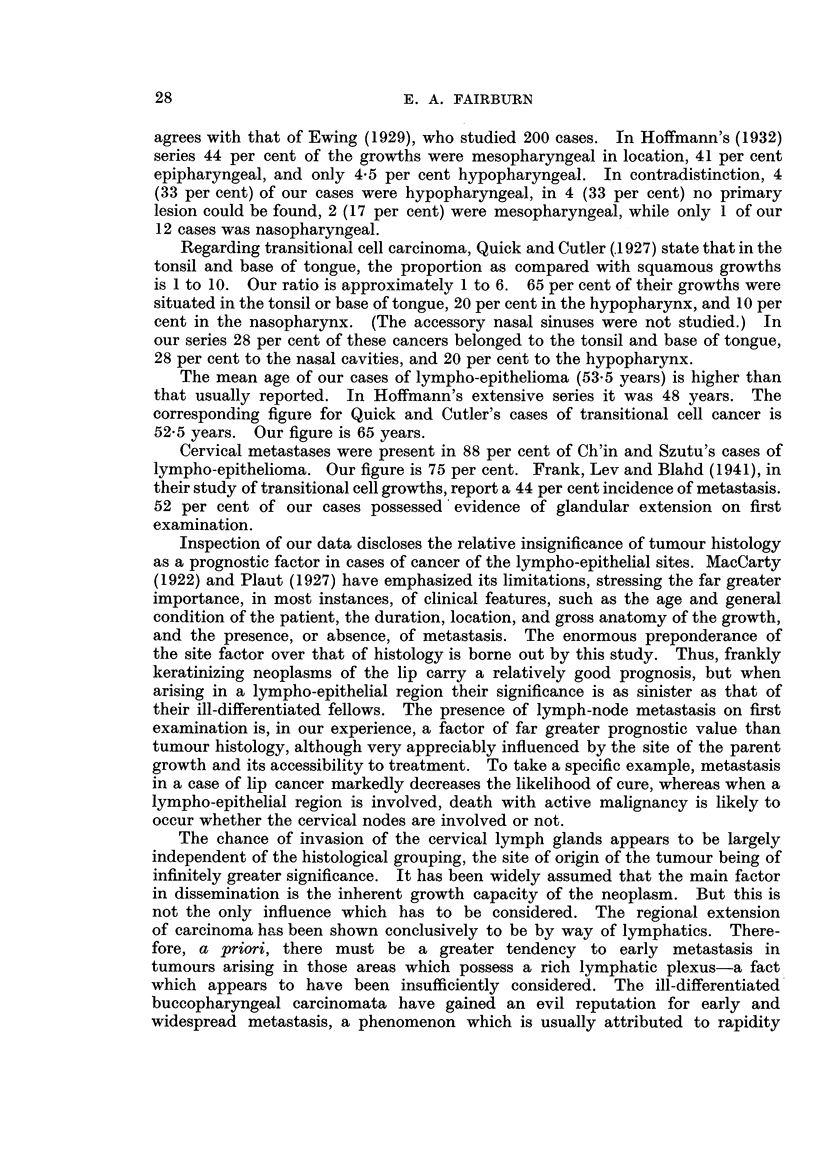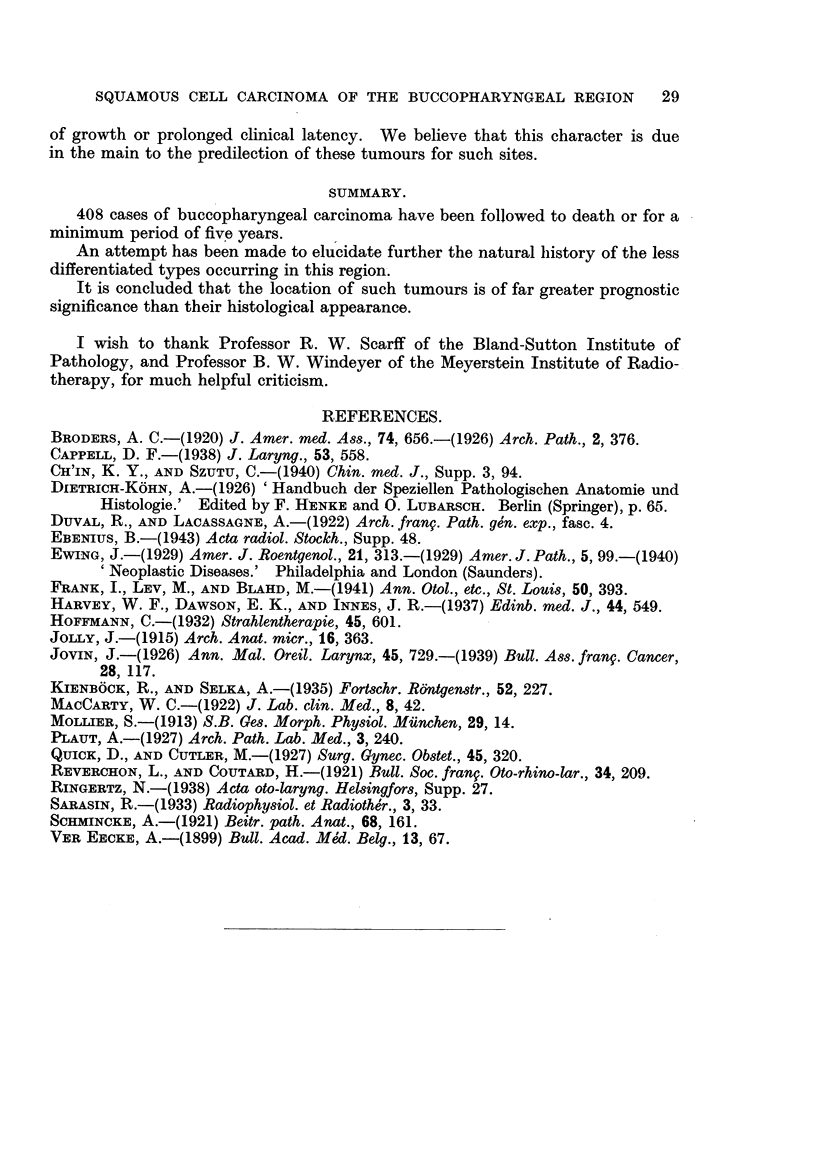# Squamous Cell Carcinoma of the Buccopharyngeal Region

**DOI:** 10.1038/bjc.1947.3

**Published:** 1947-03

**Authors:** E. A. Fairburn

## Abstract

**Images:**


### SQUAMOUS CELL CARCINOMA OF THE BUCCO-PHARYNGEAL REGION.

E. A. FAIRBURN.

*From The Bland-Sutton Institute, The Middlesex Hospital, London, W. 1.*

Received for publication February 3, 1947.

IN recent years many attempts have been made to correlate the histological appearances of new growths with their clinical behaviour and radio-sensitivity. These studies have disclosed the existence of a group of ill-differentiated highly malignant but equally highly radiosensitive buccopharyngeal carcinomata of uncertain histogenesis. While much attention has been paid to their morphology, the literature yields but scant information of value regarding the relative malignancy of these tumours. Varying nomenclature, inadequate follow-up, and an insufficiency of cases, render many of the views expressed little more than conjectures. The purpose of this paper is to elucidate further the natural history of this group. This we have attempted through an analysis of 408 cases of cancer in the neighbourhood of the mouth. The frankly keratinizing members of our series we have taken as a standard with which to compare the behaviour of our remaining histological types.

The morphological features of the more differentiated epitheliomata have been so completely evaluated that any further discussion would be superfluous. Duval and Lacassagne (1922) have probably provided the most complete classification of these growths, but made no attempt to correlate structure with potential malignancy. The most widely known study with this end in view is that of Broders (1920), who grouped the squamous cancers into four grades on the basis of cell differentiation. The anaplastic growths of Grade IV merit a brief description. They are characterized by their large, polygonal, clearly demarcated cells, which are distributed in groups of two or three, in sheets, columns, or as a diffuse infiltration. The cytoplasm is eosinophil in reaction, while the sharply cut, round or oval nucleus houses a coarse chromatin network with a prominent nucleolus. Such growths belong to that poorly differentiated group which has aroused such interest during the past two decades, a group which has become a veritable pathologists' Babel. It is a heterogeneous assembly, including transitional cell carcinoma, lympho-epithelioma, and the non-keratinizing squamous cancers which we have just described.

"Lympho-epithelioma" was the term employed by both Régaud (in a paper by Reverchon and Coutard) and Schmincke in 1921 to describe what they regarded as being a specific group of highly radiosensitive tumours arising from the so-called pharyngeal lympho-epithelium. It was Ver Eecke in 1899 who first employed the latter term ("tissu lymphothélial") in a paper concerning the histogenesis of the frog's thymus. Jolly (1915) (working particularly with the bursa Fabricii of birds) and Mollier (1913) elaborated the concept in a series of papers, extending it to all tissues characterized by a covering epithelium infiltrated by underlying lymphoid deposits. So intimate and fundamental was this relationship, they believed, that such tissues should be regarded as a class apart and designated as such. Lympho-epithelium so defined is found in the faucial tonsils, the nasopharynx, laryngopharynx, base of tongue, thymus, and ileum. Since their first description in 1921 lympho-epitheliomata have been reported as arising from all these organs with the exception of the small intestine. Tonsillar growths earlier described as alveolar sarcomata and encephaloid epitheliomata were probably of this nature (Dietrich, 1926). In 1926 Jovin published a series of cases, drawing attention to the salient clinical features of the tumour and elaborating Regaud's morphological study. Three years later a paper by Ewing further established the neoplasm as a specific entity.

The tumours occur at all ages, but particularly in middle and late adult life, males appearing to be affected more often than females. The primary lesion grows slowly and tends to be clinically latent for a considerable period of time, especially if it is situated in a region such as the nasopharynx. It appears as a smooth or finely granular, firm, globular tumour, at first a mere nodule, but later attaining considerable dimensions. Infiltrative spread and superficial ulceration occur fairly late in the evolution of the growth. Cervical lymph glandular metastases appear at an early date, while the final phase is frequently ushered in by distant visceral extension, osseous deposits being rather characteristic (Kienbock and Selka, 1935 ; Ch'in and Szutu, 1940). The histological picture may be quite typical. Syncytia of large, palely staining cells appear as sheets, irregular clumps, or columns, in a lymphocytic stroma of varying density. The cell outlines are indistinct or quite indiscernible. The nuclei are large, round or oval, vesicular, and with prominent nucleoli. The appearance described by Schmincke is somewhat different. The epithelial cells are more discrete, and in many areas lie scattered independently, while there is a heavy lymphocytic infiltration throughout. Ewing was doubtful of the specificity of Schmincke's description, and found its distinction from other types of undifferentiated pharyngeal growth difficult or impossible.

The term "Transitional Cell Carcinoma" was first employed by Quick and Cutler (1927) to describe a group of highly radiosensitive buccopharyngeal tumours of uncertain histogenesis which lacked the classical features of epithelioma, but presented transitional epithelial characters. Ewing's writings (1929, 1940) have done much to clarify the morphological features of this growth. The most frequently encountered sites are the tonsil, base of tongue, nasopharynx, laryngopharynx, nasal cavity, and accessory nasal sinuses. Quick and Cutler described the primary lesion as possessing "a finely granular, velvety surface, which looks like an erosion of the mucous membrane rather than a frank ulceration. The lesion . . . gives the impression of having originated in the deeper structures and adhered to and eroded the mucous membrane from beneath." This description is, however, far from being specific. The clinical course resembles that of lympho-epithelioma—a small and often occult primary focus, and early cervical lymph glandular metastasis. Histologically, transitional cell cancer is typified by the presence of broad, clearly demarcated, epithelial columns and alveolar formations. The cells are cylindrical, polygonal or spindle-shaped, with a rather hazy cytoplasmic membrane. The oval or fusiform nucleus is more chromatic than that of lympho-epithelioma, while its nucleolus is less distinct. Frequently the marginal cells appear regimented as though forming a basal layer. Degeneration and necrosis of the central zones of some of the epithelial formations may be observed. Lymphocytic infiltration of the stroma may be present, but is never marked.

There is considerable divergence of opinion regarding the justification of the classification of lympho-epithelioma and transitional cell carcinoma as specific entities. Ewing (1940) accepts the distinction, but admits that when atypical they may be quite indistinguishable from each other and from other ill-differentiated epithelial and mesoblastic growths. The majority of the French authors employ the term "lympho-epithelioma" to embrace both tumours (Jovin, 1939). Harvey, Dawson and Innes (1937) believe that the descriptions cover two types of growth—squamous cell carcinoma and reticulum-cell or lymphosarcoma. Cappell (1938) could not make an absolute distinction between lympho-epithelioma and transitional cell cancer.

### MATERIAL.

The material at our disposal consists of 408 cases of carcinoma of the buccal cavity, pharynx, larynx, and accessory nasal sinuses treated at the Middlesex Hospital between the years 1930 and 1939. Each has been traced to death or for a minimum period of five years following the first examination. For purposes of classification we have created the following groups according to the site of the primary lesion :

1. Upper and lower lips and commissures (purely cutaneous growths have been excluded), 2. Larynx proper. 3. Tongue (excluding base). 4. Floor of mouth. 5. Upper and lower alveoli, cheek, palate, and anterior pillars of fauces. 6. Faucial tonsil, and base of tongue. 7. Laryngopharynx, nasopharynx, and posterior oropharyngeal wall. 8. Nasal cavity and accessory nasal sinuses. 9. Cases in which cervical metastases were present in the absence of a clinically demonstrable primary lesion. We have distinguished six histological groups : I, Frankly keratinizing squamous cell carcinoma (Fig. 2 and 3) ; II, early keratinizing squamous cell carcinoma (Fig. 4 and 5) ; III, anaplastic squamous cell carcinoma (Fig. 6 and 7) ; IV, transitional cell carcinoma (Fig. 8 and 9) ; V, lympho-epithelioma (Fig. 10 and 11) ; VI, undifferentiated carcinoma (Fig. 12 and 13). Group I corresponds approximately to Broders' (1926) Grades I and II, Group II to his Grade III and part of IV, and Group III to the least differentiated residue of Grade IV. We have followed Ewing (1929) in including in Group IV the transitional-like growths arising from the pseudo-stratified columnar epithelium of the nose and accessory nasal cavities (the so-called Schneiderian carcinoma, and the solid cylindrical cell carcinoma of Ringertz (1938)). We have restricted the group, lympho-epithelioma, to tumours of the Régaud type. Our undifferentiated group corresponds to that of Ringertz. The cells are distributed in solid masses or diffuse infiltrations. The cell body is spheroidal or polygonal in form with palely staining cytoplasm, and a relatively large, oval, hyperchromatic nucleus which frequently shows bizarre, atypical features and mitotic figures.

These classifications have presented certain difficulties: 1. The situation of a tumour involving more than one of our regional groups. In such circumstances we have endeavoured to determine the original site of origin from a knowledge of the patient's history. 2. The distinction between local recurrence and new primary growth. Here we have largely followed the principles of Sarasin (1933) and Ebenius (1943). The latter recorded as a recurrence all growths which appeared 1·0 cm. or less from the periphery of the primary lesion. 3. Variation of tumour histology according to the site of biopsy. The less differentiated group has been recorded. 4. Variation of tumour histology with the passage of time. The earliest biopsy only has been noted.

### ANALYSIS OF CASES.

Table I shows the distribution of our 408 cases with regard to site and histology.

TABLE I.—*Histology and Site of the Tumours.*

| Site. | Kerat. | E. kerat. | An. sq. | Trans. | L-E. | Undiff. | Total. | Site group. | Total. | Percentage. |
|---|---|---|---|---|---|---|---|---|---|---|
| Lower lip | 37 | 4 | .. | .. | .. | .. | 41 | | | |
| Upper lip | 1 | .. | .. | .. | .. | .. | 1 | 1 | 60 | 15 |
| Lip commissure | 17 | 1 | .. | .. | .. | .. | 18 | | | |
| Vocal cord | 10 | 3 | .. | .. | .. | .. | 13 | 2 | 18 | 4 |
| Larynx (other) | 5 | .. | .. | .. | .. | .. | 5 | | | |
| Tongue (antr. ⅔) | 59 | 10 | .. | 1 | 1 | .. | 71 | 3 | 71 | 17 |
| Floor of mouth | 31 | 8 | 1 | 1 | .. | .. | 41 | 4 | 41 | 10 |
| Upper alveolus | 5 | 2 | .. | .. | .. | .. | 7 | | | |
| Lower alveolus | 7 | 6 | 1 | 2 | .. | .. | 16 | | | |
| Cheek | 7 | .. | 1 | .. | .. | .. | 8 | 5 | 63 | 16 |
| Palate | 5 | 3 | 1 | .. | .. | .. | 9 | | | |
| Antr. pillar | 17 | 3 | 2 | 1 | .. | .. | 23 | | | |
| Faucial tonsil | 12 | 17 | 2 | 6 | 2 | .. | 39 | 6 | 52 | 13 |
| Tongue (postr. ⅓) | 5 | 6 | 1 | 1 | .. | .. | 13 | | | |
| Postr. oropharynx | 2 | 1 | .. | .. | .. | .. | 3 | | | |
| Arytenoid region | 10 | 16 | 5 | 5 | 4 | .. | 40 | 7 | 54 | 13 |
| Post-cricoid region | 6 | 2 | .. | .. | .. | .. | 8 | | | |
| Nasopharynx | .. | 2 | .. | .. | 1 | .. | 3 | | | |
| Nasal cavity | .. | .. | 1 | 1 | .. | 1 | 3 | | | |
| Maxillary sinus | 8 | 3 | 3 | 6 | .. | 7 | 27 | 8 | 33 | 8 |
| Ethmoidal sinus | 1 | 1 | .. | .. | .. | 1 | 3 | | | |
| No primary found | 1 | 4 | 6 | 1 | 4 | .. | 16 | 9 | 16 | 4 |
| Total | 246 | 92 | 24 | 25 | 12 | 9 | 408 | | | |
| Percentage | 60 | 23 | 6 | 6 | 3 | 2 | | | | |

Abbreviations employed: Kerat. = Keratinizing squamous cell carcinoma. E. kerat. = Early keratinizing squamous cell carcinoma. An. sq. = Anaplastic squamous cell carcinoma. Trans. = Transitional cell carcinoma. L-E. = Lympho-epithelioma. Undiff. = Undifferentiated carcinoma.

Fig. 1 illustrates the percentage of the different types of growth in our site groups. It will be noted that 5 of our 12 lympho-epitheliomata were located in the pharynx (9 per cent of the group), and 2 in the tonsil (4 per cent of the group), while 4 were discovered in cervical metastases in the absence of a clinically evident primary lesion (25 per cent of the group). It is probable that the parent

FIG. 1.—Histology and site.

growths of the latter group were in most instances pharyngeal in location. Seven of the 25 transitional cell carcinomata arose in the tonsillar region (13 per cent of the group), a further 7 in the nasal cavities (21 per cent), and 5 in the pharynx (9 per cent). Thus, with the exception of the nasal cavities, the transitional

FIG. 2.—Frankly keratinizing squamous cell carcinoma. (× 100.)
FIG. 3.—Frankly keratinizing squamous cell carcinoma. (× 210.)
FIG. 4.—Early keratinizing squamous cell carcinoma. (× 100.)
FIG. 5.—Early keratinizing squamous cell carcinoma. (× 210.)

Fairburn.

FIG. 6.—Anaplastic squamous cell carcinoma. (× 100.)

FIG. 7.—Anaplastic squamous cell carcinoma. (× 210.)

FIG. 8.—Transitional cell carcinoma. (× 100.)

FIG. 9.—Transitional cell carcinoma. (× 210.)

FIG. 10.—Lympho-epithelioma. (× 100.)

FIG. 11.—Lympho-epithelioma. (× 210.)

FIG. 12.—Undifferentiated carcinoma. (× 100.)

FIG. 13.—Undifferentiated carcinoma. (× 210.)

Fairburn.

cell growths seem to show the same predilection for the lympho-epithelial regions as the true lympho-epithelioma. Moreover, it will be noted that it is in such sites that the incidence of the poorly differentiated squamous cancers is highest.

Table II shows the mean ages of those suffering from cancer of various histological types. It is seen that the lympho-epitheliomata and the undifferentiated

TABLE II.—*Mean Ages of the Histological Types.*

| Histology. | Kerat. | E. kerat. | An. sq. | Trans. | L-E. | Undiff. |
|---|---|---|---|---|---|---|
| Mean age in years | 61·5 | 60·5 | 61·0 | 64·5 | 53·5 | 52·5 |
| Standard deviation | 10·25 | 10·31 | 12·15 | 10·11 | 11·94 | 13·72 |

group possess the lowest mean ages (53·5 and 52·5 years respectively), while the transitional growths head the series (64·5 years). Compared with the figure for the frankly keratinizing group (61·5 years), the former two differences are statistically significant, while the latter is not. There is no significant difference between the mean ages of our cases when distributed according to the site of the primary growth. They varied from 58·5 (pharynx and nasal sinuses) to 62 years (floor of mouth and tonsil).

Table III illustrates the relationship between the histological type of the growth and the fate of the patient over a minimum period of five years. It is

TABLE III.—*Five Year Prognosis according to Histological Type.*

| Histology. | Kerat. | E. kerat. | An. sq. | Trans. | L-E. | Undiff. |
|---|---|---|---|---|---|---|
| Percentage dying with cancer | 63·5 | 92·5 | 79·0 | 72·0 | 91.5 | 78.0 |
| Median survival time in months of those dying with cancer | 9·0 | 9·75 | 10·75 | 14·5 | 9·75 | 10·5 |
| *Interquartile range in months | 6–118 | 6–15 | 6–18 | 8·25–16·75 | 6–23 | 5–27·5 |

* If a series of 100 cases be arranged in ascending order of survival time, the range of survival time from the 25th to the 75th member of the series is the interquartile range.

seen that those possessing a frankly keratinizing tumour are the least likely to die with cancer (63·5 per cent of the group die in this manner), while, at the other extreme, 92·5 per cent and 91·5 per cent of the bearers of early keratinizing cancers and lympho-epitheliomata respectively succumb with evidence of new growth. Regarding the median survival times of those dying with cancer, that of the frankly keratinizing group is shortest (9 months), and that of transitional cell cancer is longest (14·5 months). The number of cases in this group is, however, too small to draw any definite conclusion upon a statistical basis. The remaining survivals approximate to that of the frankly keratinizing group.

Table IV shows the relationship between the histology of the primary lesion and the incidence of cervical lymph glandular extension on first examination.

TABLE IV.—*Incidence of Metastasis and Effect on Prognosis.*

| Histology. | Kerat. | E. kerat. | An. sq. | Trans. | L-E. | Undiff. |
|---|---|---|---|---|---|---|
| Percentage with metastasis | 36 | 59 | 67 | 52 | 75 | 22 |
| Percentage dying with cancer among those with metastasis | 90 | 96 | 89 | 77 | 100 | 100 |
| Percentage dying with cancer amongst the metastasis-free | 49 | 87 | 62 | 67 | 67 | 71 |

We have adopted Ebenius' clinical criteria of metastasis. The frankly keratinizing cancers possess a statistically significant lower incidence of metastasis (36 per cent) than the remainder of their fellows, with the exception of the undifferentiated group which only metastasized in 22 per cent of cases. This table also illustrates the influence of glandular metastasis upon the likelihood of death with active growth. As expected, the presence of lymph nodal extension on first examination markedly increases the probability of death of this nature. If deposits are present there is a uniformly high incidence of death with cancer, ranging from 77 per cent in the case of transitional tumours to 100 per cent in the cases of lympho-epithelioma and the undifferentiated carcinomata. In the absence of metastasis the likelihood of a five-year cure is considerably increased in the case of the frankly keratinizing group, but with the other types of growth the improvement in prognosis is less impressive.

Table V is of a similar nature to Table III, but is concerned with the influence of tumour location. It is seen that 93 per cent of those falling into the pharyngeal

TABLE V.—*Five Year Prognosis according to Tumour Location.*

| Site groups. | 1 | 2 | 3 | 4 | 5 | 6 | 7 | 8 |
|---|---|---|---|---|---|---|---|---|
| Percentage dying with cancer | 35 | 55 | 68 | 73 | 78 | 90 | 93 | 82 |
| Median survival time in months of those dying with cancer | 9 | 23 | 8·5 | 12 | 11·75 | 9·75 | 7 | 12 |
| Interquartile range in months | 7–15 | 14·5–49·75 | 6–16 | 6–20 | 7–22·75 | 6–13 | 5–14 | 7–26·5 |

group and 90 per cent of the tonsillar group die with cancer, while at the other extreme is the lip, with an incidence of only 35 per cent. Regarding median survival time, that of the pharyngeal group is the shortest (7 months), and that of laryngeal cases the longest (23 months).

Table VI is the counterpart of Table IV. It shows the relationship between the site of the primary tumour and the incidence of glandular metastasis on first

TABLE VI.—*Incidence of Metastasis and Effect on Prognosis.*

| Site Groups. | 1. | 2. | 3. | 4. | 5. | 6. | 7. | 8. |
|---|---|---|---|---|---|---|---|---|
| Percentage with metastasis | 28 | 6 | 39 | 54 | 47 | 62 | 63 | 9 |
| Percentage dying with cancer amongst those with metastasis | 71 | 100 | 86 | 95 | 93 | 100 | 91 | 100 |
| Percentage dying with cancer among the metastasis-free | 21 | 53 | 56 | 47 | 64 | 80 | 95 | 81 |

examination. The pharyngeal and tonsillar groups possess the highest incidence (63 per cent and 62 per cent respectively), while the larynx and nasal cavities show the lowest (6 per cent and 9 per cent), and again it is seen that the presence of cervical metastases markedly increases the likelihood of death with cancer. The pharyngeal group provides an exception to this statement. The difference between a 91 per cent incidence of death with malignancy in those with metastasis and a 95 per cent incidence in the metastasis-free is, however, insignificant. There is a uniformly high percentage of death with cancer if extension has occurred, ranging from 71 per cent in the case of the lip to 100 per cent in the cases of the larynx and nasal cavities. As in Table IV, there is considerable inter-group variation if metastases are absent, ranging from 21 per cent in the instance of the lip to 95 per cent in the case of the pharynx.

Finally, we are considering only the lympho-epithelial sites (tonsil, base of tongue, laryngopharynx (excluding the post-cricoid region), and nasopharynx).

Table VII is similar to Tables III and V. The tendency here is towards the abolition of differences between the histological groups, the incidence of death

TABLE VII.—*Five Year Prognosis according to Histological Type. Lympho-Epithelial Regions.*

| Histology. | Kerat. | E. kerat. | An. sq. | Trans. | L-E. |
|---|---|---|---|---|---|
| Percentage dying with cancer | 85 | 100 | 75 | 75 | 100 |
| Median survival time in months of those dying with cancer | 8 | 9 | 9·5 | 11·5 | 20 |
| Interquartile range in months | 6–10 | 6–13 | 5·75–13 | 8–15 | 8–34·5 |

with cancer being 75 per cent or over in all instances. Concerning the median survival times the salient features of Table III are preserved with the exception of the longer survival of the cases of lympho-epithelioma (20 months). This is attributable to the fact that one-third of all our cases of this tumour belong to the group in which there were cervical metastases in the absence of a clinically evident primary lesion, a group which naturally carries a very low median survival time.

TABLE VIII.—*Incidence of Metastasis and Effect on Prognosis. Lympho-Epithelial Regions.*

| Histology. | Kerat. | E. kerat. | An. sq. | Trans. | L-E. |
|---|---|---|---|---|---|
| Percentage with metastasis | 52 | 68 | 62·5 | 83 | 71 |
| Percentage dying with cancer among those with metastasis | 100 | 100 | 80 | 80 | 100 |
| Percentage dying with cancer among the metastasis-free | 69 | 100 | 67 | 100 | 100 |

Table VIII should be compared with Table III. A levelling influence—tumour location—is seen at play, the range in incidence of metastasis varying only from 52 per cent for frankly keratinizing growths to 83 per cent for transitional cell cancer. It thus appears that all carcinomata arising in the lympho-epithelial regions metastasize at an early date in the clinical course of the disease whatever the histological type may be. Moreover, the presence or absence of cervical lymph glandular metastases on first examination is of little significance with regard to the likelihood of subsequent death with cancer, there being a very high incidence for all types of growth in both instances.

### DISCUSSION.

Concerning the relative incidence of lympho-epithelioma in different anatomical sites, our figures bear little resemblance to those of Ch'in and Szutu (1940), who report 97 original cases. However, our figure of 4 per cent for the tonsillar group agrees with that of Ewing (1929), who studied 200 cases. In Hoffmann's (1932) series 44 per cent of the growths were mesopharyngeal in location, 41 per cent epipharyngeal, and only 4·5 per cent hypopharyngeal. In contradistinction, 4 (33 per cent) of our cases were hypopharyngeal, in 4 (33 per cent) no primary lesion could be found, 2 (17 per cent) were mesopharyngeal, while only 1 of our 12 cases was nasopharyngeal.

Regarding transitional cell carcinoma, Quick and Cutler (1927) state that in the tonsil and base of tongue, the proportion as compared with squamous growths is 1 to 10. Our ratio is approximately 1 to 6. 65 per cent of their growths were situated in the tonsil or base of tongue, 20 per cent in the hypopharynx, and 10 per cent in the nasopharynx. (The accessory nasal sinuses were not studied.) In our series 28 per cent of these cancers belonged to the tonsil and base of tongue, 28 per cent to the nasal cavities, and 20 per cent to the hypopharynx.

The mean age of our cases of lympho-epithelioma (53·5 years) is higher than that usually reported. In Hoffmann's extensive series it was 48 years. The corresponding figure for Quick and Cutler's cases of transitional cell cancer is 52·5 years. Our figure is 65 years.

Cervical metastases were present in 88 per cent of Ch'in and Szutu's cases of lympho-epithelioma. Our figure is 75 per cent. Frank, Lev and Blahd (1941), in their study of transitional cell growths, report a 44 per cent incidence of metastasis. 52 per cent of our cases possessed evidence of glandular extension on first examination.

Inspection of our data discloses the relative insignificance of tumour histology as a prognostic factor in cases of cancer of the lympho-epithelial sites. MacCarty (1922) and Plaut (1927) have emphasized its limitations, stressing the far greater importance, in most instances, of clinical features, such as the age and general condition of the patient, the duration, location, and gross anatomy of the growth, and the presence, or absence, of metastasis. The enormous preponderance of the site factor over that of histology is borne out by this study. Thus, frankly keratinizing neoplasms of the lip carry a relatively good prognosis, but when arising in a lympho-epithelial region their significance is as sinister as that of their ill-differentiated fellows. The presence of lymph-node metastasis on first examination is, in our experience, a factor of far greater prognostic value than tumour histology, although very appreciably influenced by the site of the parent growth and its accessibility to treatment. To take a specific example, metastasis in a case of lip cancer markedly decreases the likelihood of cure, whereas when a lympho-epithelial region is involved, death with active malignancy is likely to occur whether the cervical nodes are involved or not.

The chance of invasion of the cervical lymph glands appears to be largely independent of the histological grouping, the site of origin of the tumour being of infinitely greater significance. It has been widely assumed that the main factor in dissemination is the inherent growth capacity of the neoplasm. But this is not the only influence which has to be considered. The regional extension of carcinoma has been shown conclusively to be by way of lymphatics. Therefore, *a priori*, there must be a greater tendency to early metastasis in tumours arising in those areas which possess a rich lymphatic plexus—a fact which appears to have been insufficiently considered. The ill-differentiated buccopharyngeal carcinomata have gained an evil reputation for early and widespread metastasis, a phenomenon which is usually attributed to rapidity of growth or prolonged clinical latency. We believe that this character is due in the main to the predilection of these tumours for such sites.

SUMMARY.

408 cases of buccopharyngeal carcinoma have been followed to death or for a minimum period of five years.

An attempt has been made to elucidate further the natural history of the less differentiated types occurring in this region.

It is concluded that the location of such tumours is of far greater prognostic significance than their histological appearance.

I wish to thank Professor R. W. Scarff of the Bland-Sutton Institute of Pathology, and Professor B. W. Windeyer of the Meyerstein Institute of Radiotherapy, for much helpful criticism.

REFERENCES.

BRODERS, A. C.—(1920) *J. Amer. med. Ass.*, **74**, 656.—(1926) *Arch. Path.*, **2**, 376.
CAPPELL, D. F.—(1938) *J. Laryng.*, **53**, 558.
CH'IN, K. Y., AND SZUTU, C.—(1940) *Chin. med. J.*, Supp. 3, 94.
DIETRICH-KÖHN, A.—(1926) 'Handbuch der Speziellen Pathologischen Anatomie und Histologie.' Edited by F. HENKE and O. LUBARSCH. Berlin (Springer), p. 65.
DUVAL, R., AND LACASSAGNE, A.—(1922) *Arch. franç. Path. gén. exp.*, fasc. 4.
EBENIUS, B.—(1943) *Acta radiol. Stockh.*, Supp. 48.
EWING, J.—(1929) *Amer. J. Roentgenol.*, **21**, 313.—(1929) *Amer. J. Path.*, **5**, 99.—(1940) 'Neoplastic Diseases.' Philadelphia and London (Saunders).
FRANK, I., LEV, M., AND BLAHD, M.—(1941) *Ann. Otol., etc., St. Louis*, **50**, 393.
HARVEY, W. F., DAWSON, E. K., AND INNES, J. R.—(1937) *Edinb. med. J.*, **44**, 549.
HOFFMANN, C.—(1932) *Strahlentherapie*, **45**, 601.
JOLLY, J.—(1915) *Arch. Anat. micr.*, **16**, 363.
JOVIN, J.—(1926) *Ann. Mal. Oreil. Larynx*, **45**, 729.—(1939) *Bull. Ass. franç. Cancer*, **28**, 117.
KIENBÖCK, R., AND SELKA, A.—(1935) *Fortschr. Röntgenstr.*, **52**, 227.
MACCARTY, W. C.—(1922) *J. Lab. clin. Med.*, **8**, 42.
MOLLIER, S.—(1913) *S.B. Ges. Morph. Physiol. München*, **29**, 14.
PLAUT, A.—(1927) *Arch. Path. Lab. Med.*, **3**, 240.
QUICK, D., AND CUTLER, M.—(1927) *Surg. Gynec. Obstet.*, **45**, 320.
REVERCHON, L., AND COUTARD, H.—(1921) *Bull. Soc. franç. Oto-rhino-lar.*, **34**, 209.
RINGERTZ, N.—(1938) *Acta oto-laryng. Helsingfors*, Supp. 27.
SARASIN, R.—(1933) *Radiophysiol. et Radiothér.*, **3**, 33.
SCHMINCKE, A.—(1921) *Beitr. path. Anat.*, **68**, 161.
VER EECKE, A.—(1899) *Bull. Acad. Méd. Belg.*, **13**, 67.